# Supplementary figures and images for: Aspergillus fumigatus transcription factor ZfpA regulates hyphal development and alters susceptibility to antifungals and neutrophil killing during infection
Source: PLoS Pathog. 2023 May 1;19(5):e1011152. doi: 10.1371/journal.ppat.1011152 (PMC10174577; doi:10.1371/journal.ppat.1011152)

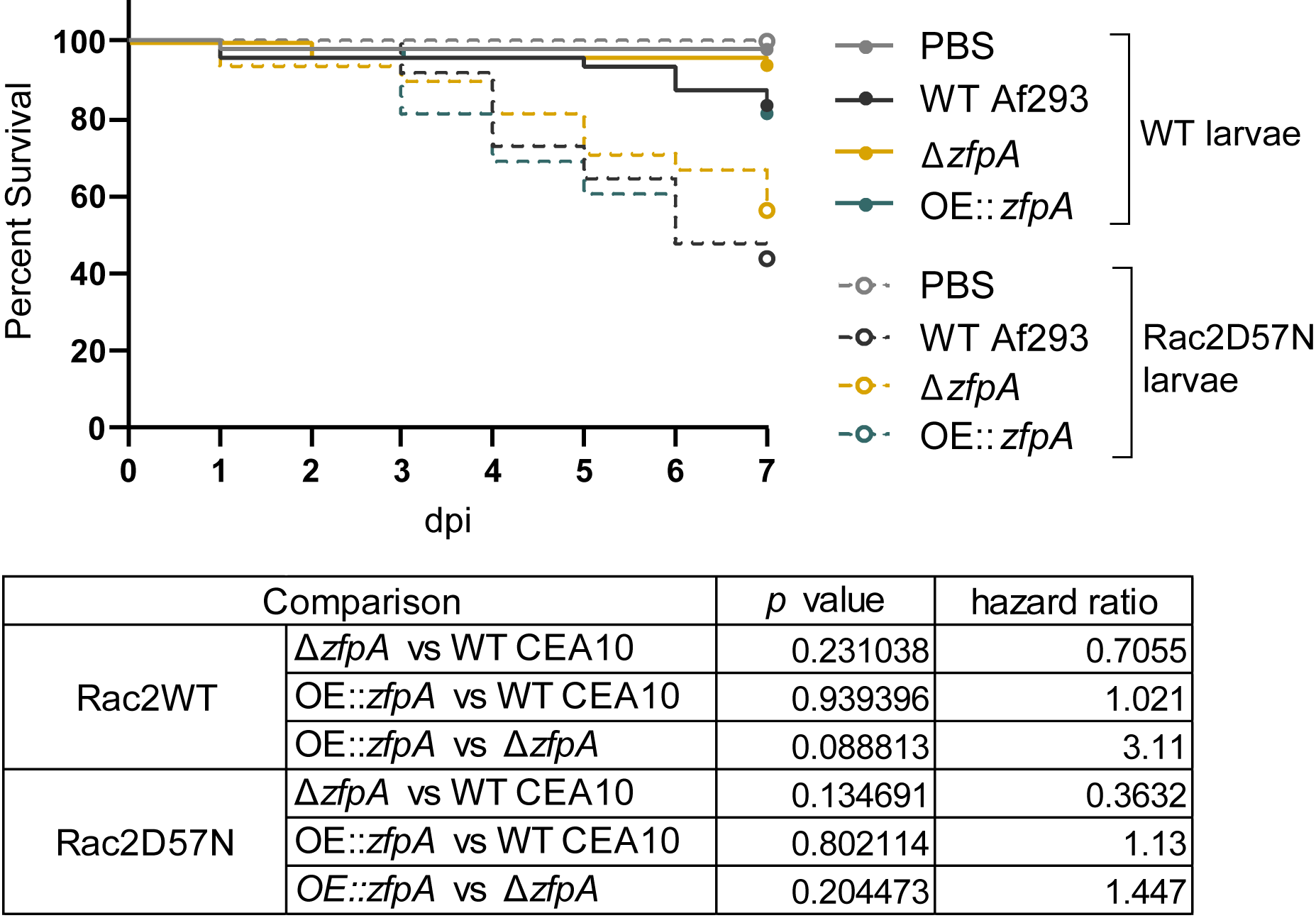

Supplement: S1 Fig — Survival analysis of larvae with the dominant negative Rac2D57N neutrophil mutation (neutrophil-deficient) or wild-type siblings injected with PBS, WT Af293, ΔzfpA, or OE::zfpA strains. WT larvae average spore dose injected: WT Af293 = 27, ΔzfpA = 29, OE::zfpA = 25. Rac2D57N larvae average spore dose injected: WT Af293 = 38, ΔzfpA = 32, OE::zfpA = 35. Results represent pooled data from 2 independent replicates. n = 46–48 larvae per condition. p values and hazard ratios calculated by Cox proportional hazard regression analysis. (TIF) [file ppat.1011152.s001.tif]

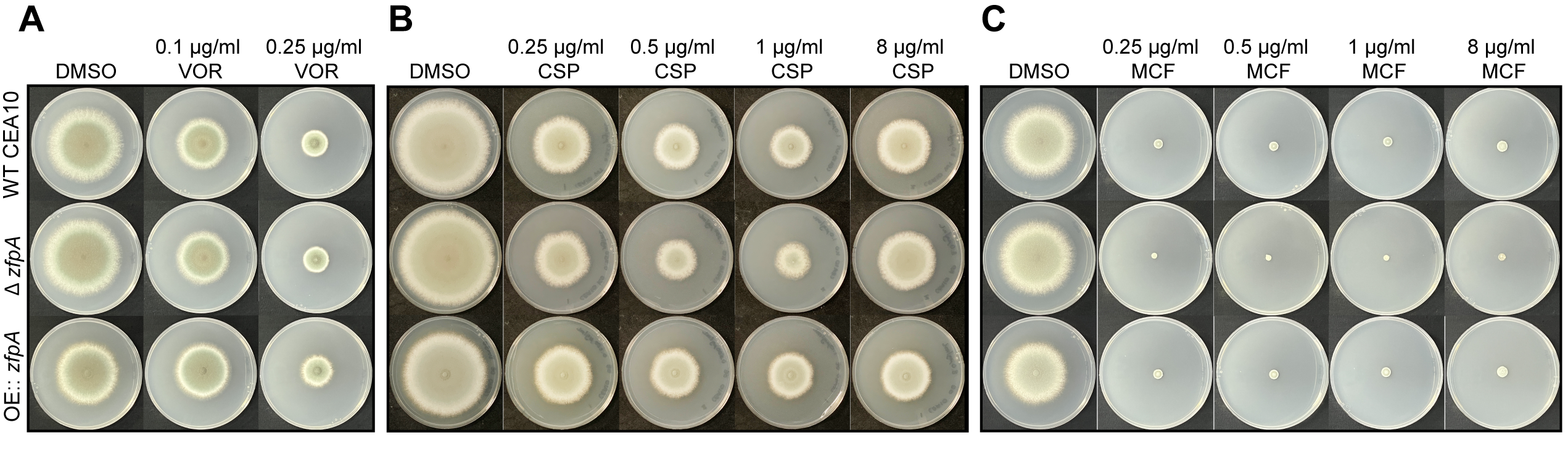

Supplement: S2 Fig — (A) Susceptibility of WT CEA10, ΔzfpA, and OE::zfpA to 0.1 and 0.25 μg/mL voriconazole (VOR). 104 spores were point-inoculated on solid GMM with voriconazole or DMSO. Images of voriconazole plates are representative of colony growth 4 days post inoculation. (B) Susceptibility of WT CEA10, ΔzfpA, and OE::zfpA to 0.25, 0.5, 1, and 8 μg/mL caspofungin (CSP). 104 spores were point-inoculated on solid GMM with caspofungin or DMSO. Images of caspofungin plates are representative of colony growth 5 days post inoculation. (C) Susceptibility of WT CEA10, ΔzfpA, and OE::zfpA to 0.25, 0.5, 1, and 8 μg/mL micafungin (MCF). 104 spores were point-inoculated on solid GMM with micafungin or DMSO. Images of micafungin plates are representative of colony growth 4 days post inoculation. (TIF) [file ppat.1011152.s002.tif]

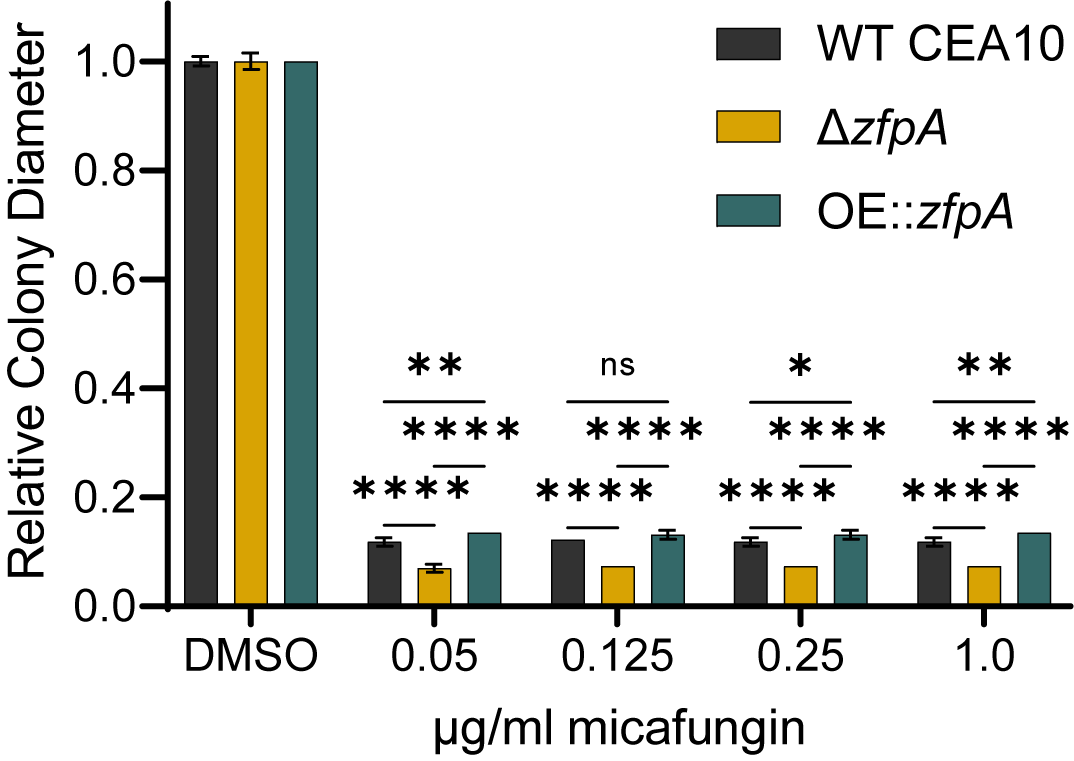

Supplement: S3 Fig — Susceptibility of WT CEA10, ΔzfpA, and OE::zfpA to 0.05, 0.125, 0.25, and 1 μg/mL micafungin. 104 spores were point-inoculated on solid GMM with micafungin or DMSO. Bars represent mean±s.d. of colony diameter at 4 days post inoculation of 4 plates per condition. p values calculated by ANOVA with Tukey’s multiple comparisons. *p<0.05, **p<0.01, ****p<0.0001. (TIF) [file ppat.1011152.s003.tif]

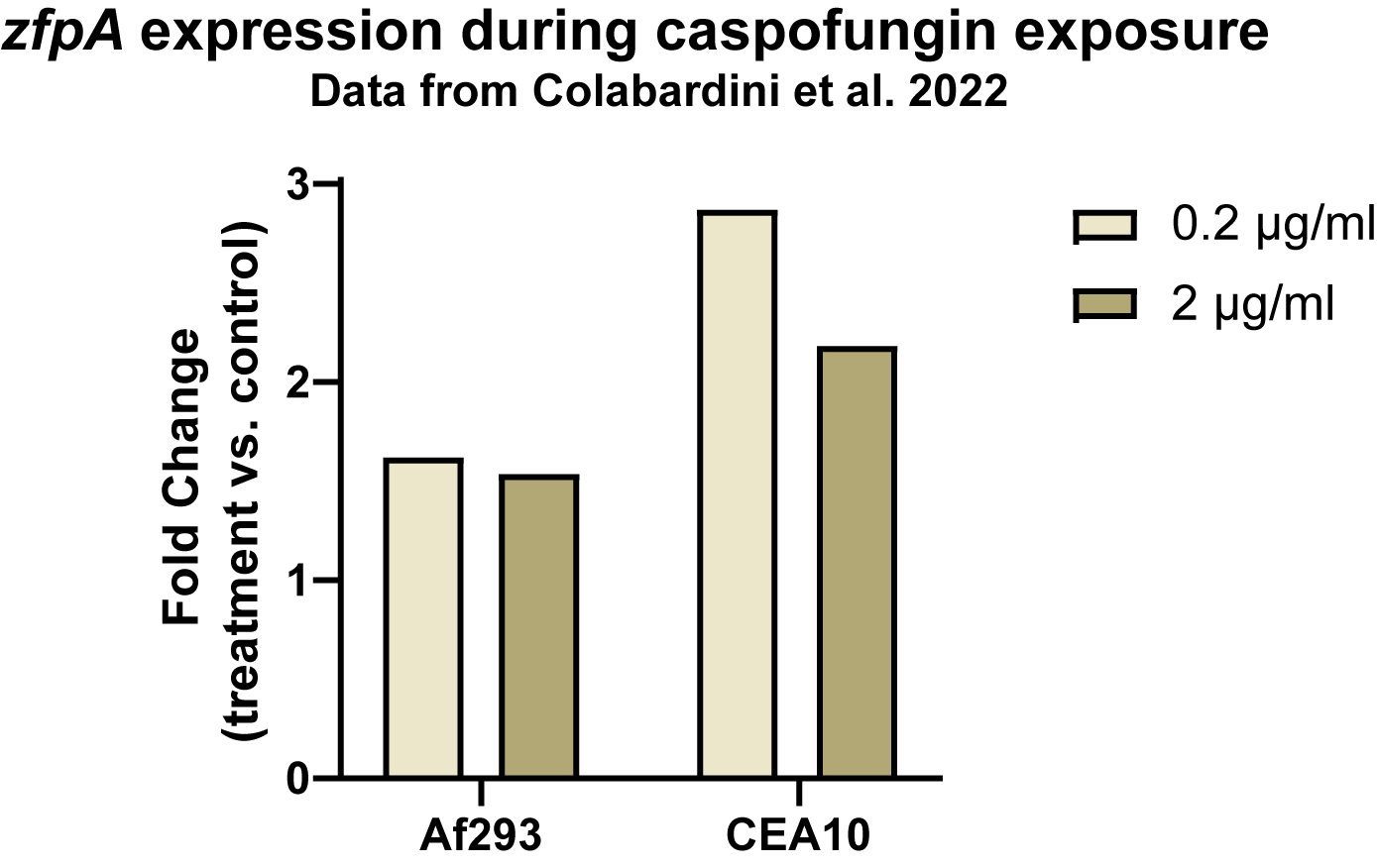

Supplement: S4 Fig — Bars represent fold change in zfpA expression in Af293 and CEA10 backgrounds during exposure to 0.2 and 2 μg/ml caspofungin. Fold change values were collected from RNAseq dataset in supplementary materials of Colabardini et al., 2022 [15]. (TIF) [file ppat.1011152.s004.tif]

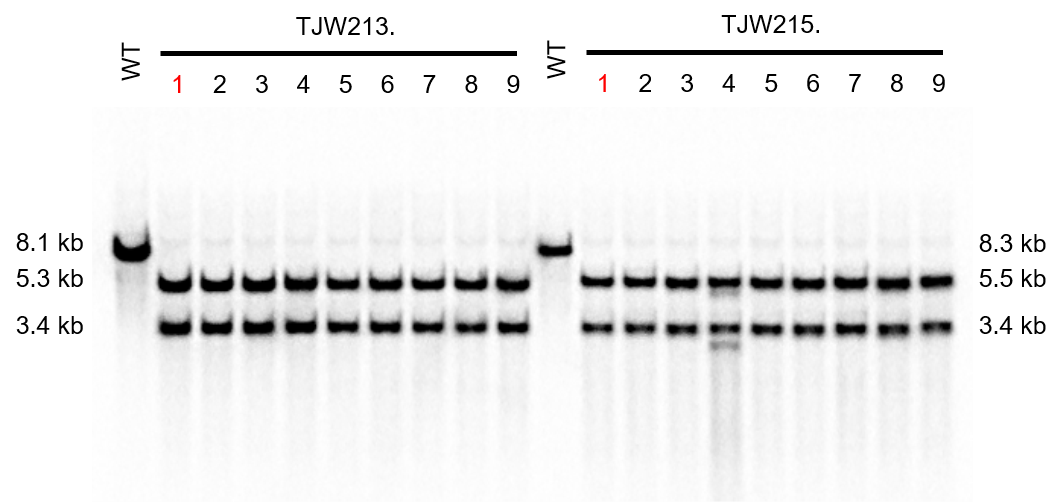

Supplement: S5 Fig — Genomic DNA was digested by PciI. Wildtype (8.1 kb for Af283, 8.3 kb for CEA10), and ΔzfpA (5.3 and 3.4 kb for Af293, 5.5 and 3.4 kb for CEA10). TJW213.1 and TJW215.1 were chosen for subsequent experiments. (TIF) [file ppat.1011152.s005.tif]

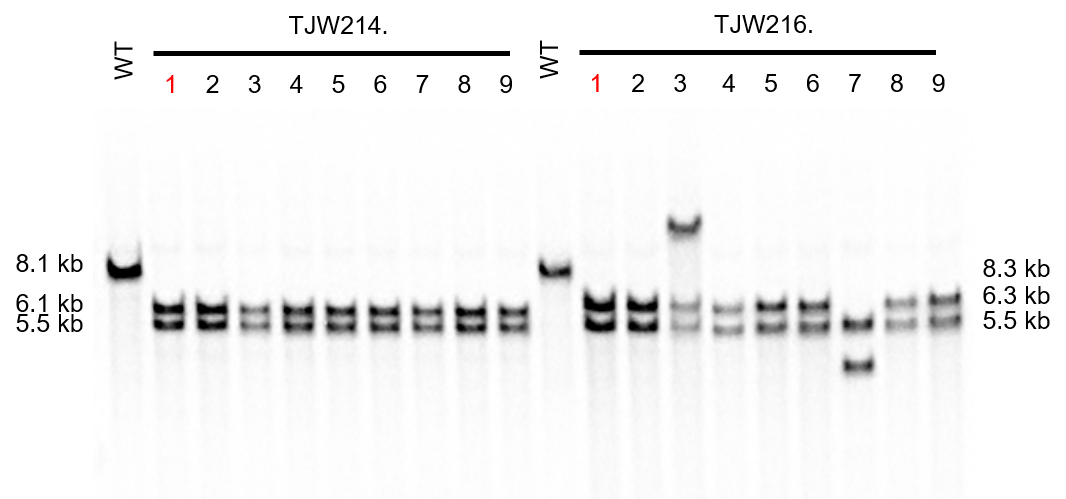

Supplement: S6 Fig — Genomic DNA was digested by PciI. Wildtype (8.1 for Af293, 8.3 kb for CEA10), and OE::zfpA (6.1 and 5.5 kb for Af293, 6.3 and 5.5 kb for CEA10). TJW214.1 and TJW216.1 were chosen for subsequent experiments. (TIF) [file ppat.1011152.s006.tif]
