# Supplementary material for: Aspergillus fumigatus transcription factor ZfpA regulates hyphal development and alters susceptibility to antifungals and neutrophil killing during infection
Source: PLoS Pathog. 2023 May 1;19(5):e1011152. doi: 10.1371/journal.ppat.1011152 (PMC10174577; doi:10.1371/journal.ppat.1011152)
Supplement: Attachment — Submitted filename: Response to Reviews.docx [file ppat.1011152.s014.docx]

We would like to thank the reviewers for their attention and thoughtful suggestions. We have addressed reviewer comments below and feel our manuscript is much improved and now suitable for publication in *PLOS Pathogens*.

Reviewer's Responses to Questions

**Part I - Summary**
Please use this section to discuss strengths/weaknesses of study, novelty/significance, general execution and scholarship.

Reviewer #1: In this study, the authors characterize the role of the Aspergillus fumigatus transcription factor ZfpA in resistance to neutrophil attack and antifungal treatment. They utilize a zebrafish infection model to show that this transcription factor is crucial in virulence because it protects hyphae against attack by neutrophils. Further, infections in neutrophil-defective zebrafish demonstrate that the zfpA mutant is exquisitely hypersensitive to the echinocandin caspofungin during infection, in contrast to a lack of hypersensitivity to voriconazole. This is a rigorous study reporting potentially important new connections among fungal cell wall structure, antifungal resistance and host-pathogen interaction. The use of the zebrafish model provides important context for the activity of ZfpA during infection and therapeutic drug treatment.

Reviewer #2: This manuscript by Schoen et al examines the outcomes of infection with a ZfpA mutant in a zebrafish model. The authors nicely show that in WT zebrafish the ZfpA is less virulent and this correlates with enhanced susceptibility to PMN antifungal killing activity. However, the authors fail to link what changes the susceptibility of the ZfpA mutant to PMN killing and what antifungal effector mechanisms from the PMN cells are utilized. Rather the authors go on to explore the cell wall integrity pathway and susceptibility to the antifungal drugs particularly chitin synthase inhibitors. They demonstrate that ZfpA mutant is more susceptible to caspofungin.

Reviewer #3: 
The authors recently reported that the transcription factor ZfpA has an important role in hyphal development. In the current study, these investigators use a ΔzfpA mutant to demonstrate the importance of ZfpA in virulence and in resistance to immunity in a zebrafish model of infection and in response to human neutrophils. Further, the use of well characterized anti-fungal agents identify a specific role for ZfpA in chitin synthesis. Overall, the data as presented are clear and in general support the conclusions. However, there are a few points that the authors should address.

**Part II – Major Issues: Key Experiments Required for Acceptance**
Please use this section to detail the key new experiments or modifications of existing experiments that should be absolutely required to validate study conclusions.

Generally, there should be no more than 3 such required experiments or major modifications for a "Major Revision" recommendation. If more than 3 experiments are necessary to validate the study conclusions, then you are encouraged to recommend "Reject".

**Reviewer #1:** None.

**Reviewer #2:** Experimentally the authors nicely demonstrated that WT zebrafish infected with the ZfpA are less susceptible to infection (Figure 1A) and this correlates with enhanced susceptibility to PMN antifungal killing activity (Figure 3B/C).

1. Why is the ZfpA mutant more susceptible to PMN killing? Do the cell wall alterations change the ability of the fungal germlings to induced PMN activation?

2. What antifungal effector mechanisms from the PMN cells are utilized to control the ZfpA mutant? Are phox-deficient PMNs unable to kill the ZfpA mutant germlings or is an alternative mechanism (PMN degranulation or NETosis)?

Thank you for these suggestions, we have assessed multiple neutrophil effector functions in our revised manuscript (Figs 3-4). In figure 3, we have added an additional analysis of the timing of neutrophil contact with germlings, and found no differences in the time it takes for neutrophils to attack the ZfpA mutants compared to WT. In figure 4, we tested whether the ZfpA mutants differentially activate neutrophils by measuring NETosis, IL-8 production, and myeloperoxidase release in response to each strain. We found no differences between strains in these assays, suggesting that neutrophil activation responses do not differ between WT and the ZfpA mutants. Additionally, to test whether the enhanced susceptibility of ∆*zfpA* is dependent on neutrophil ROS, we utilized the *p22^-/-^* larval zebrafish model which lacks ROS production in phagocytes (Schoen et al., 2019). We found that virulence of ∆*zfpA* is still attenuated compared to WT CEA10 in the *p22* mutants. This result, along with our *in vitro* assessment of H_2_O_2_ susceptibility, indicate that ∆*zfpA* succumbs to non-oxidative neutrophil killing mechanisms, possibly as result of weakened hyphal structural integrity.

**Reviewer #3:**

1. Fig 2,3. Quantification of the area with hyphae is certainly one method of showing total hyphae. However, although they state that loss of RFP implies killing, unless I am mistaken, the presence of RFP does not address viability. Can the authors use CFU or another method in addition imaging, which would strengthen their conclusions? Also, in addition to showing percent RFP, the authors should also show RFP values.

Our *in vivo* imaging experiments in Fig 2 use the 2D area of visible RFP signal as a measure of fungal burden. The images are analyzed by manually thresholding the RFP signal to eliminate background and only capture hyphal signal. We acquire images once every 24 hours, which provides valuable longitudinal data that allows us to see changes in fungal burden over time, where a decrease in signal area is indicative of fungal clearance. This technique has been reliably used to assess fungal burden in previous publications from our group and others (Schoen *et al*., 2019; Thrikawala *et al*., 2022; Rosowski *et al*., 2018; Seman *et al.*, 2018).

Our *in vitro* imaging experiments in Fig 3 provide more precise information on the timing of fungal killing to supplement the fungal burden data in Fig 2. Germlings were scored as dead or alive based on visible RFP signal, which can be observed in Movies S1-S3. Expression of RFP in these strains is controlled by the promoter of GpdA, an enzyme essential for glycolysis and primary metabolism. Given the importance of *gpdA* expression for cell function, we feel that the permanent loss of RFP signal and germling growth is most likely a result of cell death. We have also added supplemental movies that show loss of RFP signal following caspofungin treatment and cell lysis (supplemental movies S5-7).

2. Fig 4. The authors state that there were no visible differences between the colonies of Aspergillus strains grown in sorbitol or hydrogen peroxide. However, the examples they give appear to have differenced in the colonies of the OE::zfpA (less filamentation on the edges) when compared with WT or ΔzfpA strains at the 103 and 102 inoculums. The authors should show quantitative data based on repeat experiments.

We appreciate your attention to detail regarding colony morphology and have updated the text to highlight differences in morphology between the ZfpA mutants. Dilution plating is a standard method in the field and one that is usually presented without formal quantification, therefore we think our figure is appropriate.

3. Fig 5, 6. In contrast to Figure 4, here they state that there are differences in colony diameter. However, in the examples shown, this differences are not apparent. Overall, the agar plate results shown in Fig5A and Fig6A,C are unconvincing.

We recognize that the observed differences in relative colony diameter are small, but they are highly consistent among biological and technical replicates. The ZfpA deletion mutant has a larger radial growth than WT CEA10, while the OE::zfpA mutant has a tighter, more compact colony morphology that can be seen in the DMSO images in Fig 5-6. We account for this in our analysis by reporting colony diameter of drug treated conditions normalized to the growth of that strain on GMM+DMSO. Further, our analysis of caspofungin susceptibility using the viability reagent PrestoBlue in Fig 7 phenocopies our radial growth data.

4. Fig 7. The authors claim that the susceptibility of the ΔzfpA is due to reduced baseline chitin levels. While microscopy in Fig7A supports this conclusion, they need to quantify CFW intensity.

Thank you for this suggestion, we have added quantification of CFW signal (mean gray value) to Fig 7.

**Part III – Minor Issues: Editorial and Data Presentation Modifications**
Please use this section for editorial suggestions as well as relatively minor modifications of existing data that would enhance clarity.

**Reviewer #1:** 
1) It is not clear whether the role of ZfpA is direct or indirect in regulation of chitin levels

We agree and are actively investigating the genetic pathways under ZfpA control. Upcoming work from our lab aims to find whether ZfpA controls expression of these genes through direct DNA-binding or through interaction with other regulatory proteins.

2) It is not directly demonstrated if increased chitin is the specific causative factor in increased echinocandin resistance. Does ZfpA control other aspects of physiology that promote resistance/tolerance to echinocandins?

ZfpA also regulates hyphal branching and septation. We suspect that increased septation in the OE::*zfpA* strain partially contributes to its enhanced resistance by protecting more hyphal compartments from lysis and describe our thoughts in the discussion.

3) Please spend more space describing previous work on ZfpA and explain how this study improves upon that previous work—relative to susceptibility to antifungals and immune attack (Ref 15-18)

Thank you for this suggestion. We have expanded our discussion of previous ZfpA literature throughout the manuscript.

4) It would be interesting to see if the results with neutrophil-defective fish are the same or different from corticosteroid-treated fish, to examine different types of immunocompromise that are associated with IA. This seems like a model that has been used before in zebrafish

Dexamethasone has been successfully used with zebrafish larvae, however, we decided to focus revision experiments on more specific mechanisms of impaired immunity such as loss of phagocyte oxidase activity (Fig 4).

5) It would be useful to examine b-glucan and chitin exposure in the zfpA and OEX-ZFPA strains, as well as immune cytokine responses

A more comprehensive cell wall analysis of the ZfpA mutants is currently the focus of another graduate student for a separate study, therefore, we decided to focus on neutrophil effector functions in this paper. We’ve updated Figure 3 and have added an additional figure (Fig 4) where we assessed NET formation, neutrophil activation, cytokine production, degranulation, and the relevance of ROS in host survival.

6) The % of germlings alive was quantified in experiments with human neutrophils in vitro, providing a good measure for resistance to immune attack. How much does survival of zfpA germlings increase in neutrophil-defective fish? Also, it seems like another relevant measure would be the rate of hyphal segment death. Is it possible to measure this in time-lapse or time-course experiments in fish?

Capturing germling survival or hyphal segment death in the fish would be a very interesting experiment, but we don’t think the in vivo imaging currently has the resolution to consistently capture these events. Death of larvae is associated with a large fungal burden and given the similarity of larval survival with our mutant strains, we suspect the fungal burden to be similar as well.

7) It would be useful to have a more nuanced discussion of the idea forwarded in the Discussion that these results suggest combining chitin inhibition and septation inhibition would be an effective drug combination. Since chitin inhibition with CFW (in the context of high calcium) increases ECH resistance, combinations of cell wall inhibitors may not always yield the desired synergies—due to inherent compensatory programs.

Thank you for your insights on this, we have altered our discussion of this point in the text.

8) Please explain what PrestoBlue is measuring, and comment on the correlation between PrestoBlue activity and loss/retention of cytoplasmic fluorescence

PrestoBlue is a cell viability reagent that relies on cellular metabolic activity. The reducing environment of a live cell will lead to conversion of rezazurin to fluorescent resorufin. PrestoBlue functions similarly to XTT or MTT, but does not require addition of a substrate. PrestoBlue has been successfully used to assess viability of *Candida* spp. (Horton *et al*., 2021). We have added further explanation of the PrestoBlue assay in the results section.

We have not formally assessed the relationship between PrestoBlue signal and loss of cytoplasmic RFP, however, we do feel they are both robust measures of cellular viability. For example, the PrestoBlue viability phenotypes presented in Fig 7 phenocopy the radial growth data presented in Fig 6 and the survival outcomes in Fig 8. Regarding the loss of cytoplasmic RFP signal, we have observed loss of signal during *A. fumigatus* cell lysis during caspofungin exposure (see additional supplemental movies S5-7). Further, expression of RFP in these strains is controlled by the promoter of GpdA, an enzyme essential for glycolysis and primary metabolism. Given the importance of *gpdA* expression for cell function, we feel that the permanent loss of RFP signal and germling growth is most likely a result of cell death.

9) Please speculate on your expectations for the relative activity of ZfpA in control of these phenotypes in other isolates of Af, such as Af293.

We believe ZfpA has a similar role in Af293. We have deleted and overexpressed ZfpA in the Af293 background and observed similar survival phenotypes as were seen in the CEA10 strains. We have added a survival analysis of wild-type and Rac2D57N (neutrophil-deficient) larvae infected with ZfpA mutants in the Af293 background (S1 Fig). We have also observed similar changes in caspofungin susceptibility in the Af293 mutants.

10) Does CFW/CaCl2 treatment alter ZpfA expression or activity?

This is an interesting question, but currently we can only speculate about ZfpA activity under these conditions. Our data in Fig 7 suggest that ZfpA activity is not needed for chitin synthesis during caspofungin- or CFW/CaCl2-induced stress. However, existing transcriptome analyses of *A. fumigatus* during exposure to caspofungin (Colabardini *et al*., 2022; Valero *et al.,* 2022), voriconazole (da Silva Ferreira *et al.,* 2006), and high calcium (Malavazi *et al*., 2009) suggest that ZfpA expression is increased during these conditions. Future work from our lab aims to uncover further details of ZfpA-regulated processes.

11) In line 194, it seems like the nomenclature delta-zfpA would be more appropriate (typo)

Thank you for your attention to detail, we’ve carefully reviewed the manuscript for these types of mistakes and made the appropriate changes.

**Reviewer #2:** (No Response)

**Reviewer #3:** none
